# 肺癌脑膜转移癌诊疗的研究进展

**DOI:** 10.3779/j.issn.1009-3419.2022.102.25

**Published:** 2022-07-20

**Authors:** 乃升 高, 涛 信

**Affiliations:** 150081 哈尔滨，哈尔滨医科大学附属第二医院肿瘤内科 Department of Oncology, The Second Affiliated Hospital of Harbin Medical University, Harbin 150081, China

**Keywords:** 脑膜转移癌, 肺肿瘤, 脑脊液细胞学, 诊断, 治疗, Leptomeningeal metastases, Lung neoplasms, Cerebrospinal fluid cytology, Diagnosis, Treatment

## Abstract

脑膜转移癌（leptomeningeal metastases, LM）是晚期肺癌中进展迅速、预后差的常见并发症。脑膜转移癌临床诊断率低，治疗手段有限，疗效差，自然生存时间短。脑脊液细胞学作为诊断脑膜转移癌的金标准，但是目前临床症状及影像学阳性的患者脑脊液首次细胞病理学阳性诊断率一般不超过50%，导致脑膜转移癌患者的诊断延迟、治疗延误。随着驱动基因阳性肺癌靶向治疗、驱动基因阴性肺癌免疫治疗的进展，肺癌患者生存期延长，但是脑膜转移癌发病率逐年增加，如何提高诊断率、寻找有效的治疗手段是目前临床研究的热点。本文就肺癌脑膜转移癌诊断及治疗研究进展予以综述。

## Abstract (en)

Leptomeningeal metastases (LM), a special type of metastasis in advanced lung cancer, is known for its severe clinical symptoms, rapid progression and poor prognosis. LM used to be featured with low clinical diagnosis rate, limited treatment options, poor treatment efficacy, and very short survival if treatment not given. Though cerebrospinal fluid (CSF) cytology remains to be the gold standard for the diagnosis of LM, the positive rate of the first CSF cytology even in patients with suggestive clinical symptoms and positive imaging generally does not exceed 50%, leading to a delay in the diagnosis and treatment of patients with LM. With the progress of targeted therapy for driver gene-positive lung cancer and immunotherapy for driver gene-negative lung cancer, the overall survival of patients with lung cancer has been prolonged, meanwhile incidence of LM has been increasing year by year. Current clinical research in this field center around how to improve diagnosis rate and to find effective treatment approaches. This paper reviews advances in diagnosis and treatment of LM of lung cancer..

脑膜转移癌（leptomeningeal metastases, LM）是晚期肺癌中严重的致死性并发症。近年来由于表皮生长因子受体酪氨酸激酶抑制剂（epidermal growth factor receptor tyrosine kinase inhibitors, EGFR-TKIs）等的推广应用，肺癌患者生存期延长，但是脑膜转移癌患者发病率逐年增加^[1]^，*EGFR*突变型非小细胞肺癌（non-small cell lung cancer, NSCLC）患者的脑膜转移率可以高达10%，在尸检比例更高，确诊后未进行相关治疗的脑膜转移癌患者中位生存期（median overall survival, mOS）仅为4周-6周^[2]^。由于其诊断困难，治疗效果不佳，患者生存时间短，脑膜转移癌的诊疗成为目前研究热点^[3]^。为提高脑膜转移癌的诊断效率、寻找积极的治疗方法，现对肺癌脑膜转移癌的诊断及治疗研究进展予以综述。

## 1 肺癌脑膜转移癌的诊断

肺癌脑膜转移癌的诊断依据在肺癌确诊基础之上，基于临床表现、影像学检查、脑脊液细胞学检测进行诊断，脑脊液基因检测可以辅助诊断。

### 1.1 临床表现和影像学检查

临床表现上，由于肿瘤细胞在软脑膜中的播散，导致脑脊液循环受阻，因而产生颅内压增高以及大脑/脑膜损害等相应表现，但是表现并不特异，如：头痛、恶心、呕吐、视觉障碍、听力损失和神经认知障碍等^[4]^。影像学方面，有研究^[5]^表示磁共振成像（magnetic resonance imaging, MRI）对脑膜转移癌的敏感性为70%-87%，特异性为75%-94%，增强MRI可以提高对主要或仅表现为累及颅神经的脑膜转移癌的敏感性，其特征性表现为脑膜强化^[6]^。但在目前具有脑膜转移症状阳性的患者中，影像学阳性率不足20%，并且值得关注的是，脑膜转移癌影像学一经确诊提示预后极差^[7]^。

### 1.2 脑脊液细胞学

脑脊液细胞学检查是脑膜转移癌诊断的金标准，但是初始临床症状及影像诊断为脑膜转移癌的患者，通过腰椎穿刺行脑脊液细胞病理学检测阳性率并不高，检索相关文献^[8]^显示目前临床首次检查阳性率不超过45%，连续多次腰椎穿刺检查可以提高脑脊液阳性率，但是不超过80%。对于提高脑脊液细胞学阳性率，目前认为脑脊液样本量是影响细胞病理学检测的重要因素，应提取至少5 mL-8 mL脑脊液进行细胞学分析；脑脊液中的蛋白在离体后会迅速降解，离体后应立即进行脑脊液标本固定；可以采用腰椎穿刺或者Ommaya囊获取脑脊液标本；对初始细胞学为阴性的标本需要连续多次动态检查同时辅助脑脊液生化及基因检测，通过以上相关手段可以辅助提高脑膜转移癌的诊断^[9]^。

### 1.3 脑脊液薄层液基细胞学

薄层液基细胞学（liquid-based cytology, LBC）技术已在大多数检测细胞学标本中广泛使用。据报道，它具有更高的细胞回收率和更好的细胞保存作用^[10, 11]^。此外，已经证明LBC玻片中的细胞表达比细胞离心涂片制剂中更明显，并且LBC与细胞离心涂片法在检测异常细胞方面具有较好的敏感性和特异性^[12, 13]^。Patrizia等^[14]^研究表明细胞离心涂片和LBC在鉴别脑脊液恶性肿瘤细胞中，LBC的阳性预测值高于细胞离心涂片法（100% *vs* 95%），并且LBC法提供了更清晰的细胞背景环境，更多的细胞富集，以及更好的细胞核细节，残留液体也可在如免疫组织化学评估和分子检测中继续应用。

### 1.4 脑脊液免疫细胞化学

免疫细胞化学是肿瘤患者细胞学辅助检查的方法之一^[15]^。脑脊液细胞形态学的完整性和可视化质量是检测的重要因素。体液中的细胞在体外往往会快速降解并失去免疫反应性，对于脑脊液的低张环境来说也是如此，因此快速固定和相应的处理尤为重要。最常见的固定剂是使用100%甲醇或4%缓冲多聚甲醛，这种固定制剂在冷藏时通常能保持几天的免疫反应性，但是长期储存（数周或数月）可能会将免疫反应削弱^[16]^。Dušková1等^[17]^在提高识别脑脊液中肿瘤细胞的研究中，通过快速送检、处理样本后，根据细胞形态进一步行免疫细胞化学检查，使肿瘤细胞能最大化被识别，试验中应用甲醇固定方案并冷冻储备制剂，并对部分样本行甲醇固定后使用聚乙二醇涂覆制剂，但诊断结果较前并无明显差异^[18]^。

### 1.5 脑脊液基因检测

由于脑膜转移癌脑脊液细胞学诊断敏感性尚待完善，“液体活检”也成为目前研究的热门话题。脑膜转移癌患者脑脊液中富含肿瘤细胞、核酸及蛋白，是目前脑膜转移癌诊断的最佳“液体活检”样本，可以进行诸如游离的循环肿瘤DNA（cell free DNA, cfDNA）、循环肿瘤细胞、外泌体等相关检测。研究^[19]^表明，驱动基因阳性肺癌脑膜转移癌患者进行连续动态细胞学及基因组学检测可以有效地预测肿瘤进化谱，研究脑膜转移癌发生机制、耐药机制并对治疗判断预后有明确的指导意义。吴一龙教授团队采用NGS技术检测NSCLC脑膜转移癌患者的原发肿瘤、脑脊液和血浆。脑脊液cfDNA驱动基因检出率为100%（26/26）明显高于其他标本，与血浆和原发肿瘤组织相比，脑脊液样本显示了治疗过程*EGFR*突变型肺癌细胞呈现不同的进化图谱，该结论显示脑脊液中cfDNA可以揭示脑膜转移癌的独特遗传特征，脑脊液可被认为是*EGFR*突变型NSCLC中脑膜转移癌最有代表性的液体活检介质，提示脑脊液活检在LM诊断中的重要作用，但临床广泛应用受到限制^[20, 21]^。

## 2 肺癌脑膜转移癌的治疗

### 2.1 靶向治疗

靶向治疗为驱动基因突变型肺癌患者带来长期生存，但长期生存也使得脑膜转移癌的发生率增加^[22-24]^，如何提高驱动基因突变肺癌患者脑膜转移癌治疗疗效，成为国际临床研究的热点。

#### 2.1.1 EGFR-TKIs

在全球范围内，*EGFR*突变NSCLC患者中脑膜转移癌的发病率约为10%^[25]^，积极治疗后仅将生存期延长至3个月-11个月，虽然具有*EGFR*基因突变患者的生存期较差，但有研究^[26]^表明经治疗后与无*EGFR*突变患者相比，有44%患者的生存期延长了6个月以上，证实*EGFR*突变的脑膜转移癌患者通过治疗后预后有改善的趋势。有研究^[27]^认为*EGFR*突变出现在癌变的早期，甚至可能与脑转移倾向有关。一项回顾性研究^[28]^表明，与NSCLC患者存在*EGFR*突变相比EGFR野生型更容易发生脑转移和软脑膜播散。

厄洛替尼和吉非替尼作为第一代EGFR-TKIs，在脑膜转移癌的治疗中均展示出一定的疗效。相关研究^[29]^证实，在脑脊液渗透率方面厄洛替尼优于吉非替尼。标准剂量的吉非替尼在脑脊液中药物浓度不足^[30-32]^，因此增加EGFR-TKIs剂量可视为重要方法^[33, 34]^。研究^[35]^显示，57%的NSCLC脑膜转移癌患者的神经系统症状在服用高剂量的吉非替尼（每天750 mg或1, 000 mg）后较前缓解。另一项回顾性分析中结果并不显著。

阿法替尼是第二代EGFR-TKIs，对治疗脑转移和脑膜转移癌也有相应疗效。在两项随机Ⅲ期临床试验^[36]^的联合亚组分析中，与全身化疗相比，无症状脑转移患者的无进展生存率显著提高（8.2个月 *vs* 5.4个月）。Hoffknecht等^[37]^的研究中，存在中枢神经系统转移的患者到治疗失败时的中位生存期为3.6个月，与无中枢神经系统转移患者相比无差异。但是其中1例疗效显著的患者数据显示，阿法替尼在脑脊液中的浓度接近1 nmol/L，可渗透到中枢神经系统，因此，对于*EGFR*突变或EGFR-KIs敏感的NSCLC和中枢神经系统转移患者，阿法替尼可能是一种有效的治疗方法。

奥希替尼作为第三代口服EGFR-TKIs，可用于*EGFR* T790M突变患者^[38]^。临床数据^[39, 40]^显示，与吉非替尼、厄洛替尼或阿法替尼相比，奥希替尼的血脑屏障穿透率更高，是治疗脑膜转移癌的有效方法之一。BLOOM研究^[41]^表明初始TKIs靶向治疗发生肺癌脑膜转移癌后采用第三代靶向TKIs治疗可以延长中位无疾病进展生存时间到8.6个月左右，客观缓解率为41%-62%，显示出较好的疗效，但由于缺乏后线治疗手段，总生存期不超过12个月。虽然以上药物均对脑膜转移癌的治疗显示出良好的疗效，但在临床工作中，由于患者存在个体差异，最佳用药时间、顺序以及更换药物时间还需进一步研究。

#### 2.1.2 间变性淋巴瘤激酶（anaplastic lymphoma kinase, ALK）抑制剂

*ALK*融合基因阳性的NSCLC发生脑膜转移癌的概率约为5%。ALK抑制剂是此类患者有效的系统性治疗手段；克唑替尼是第一代ALK抑制剂，已被批准用于*ALK*重排的NSCLC患者的一线治疗。虽然克唑替尼的脑脊液穿透性较低（脑脊液与血浆的比值为0.026），但在两项随机临床研究^[42, 43]^的回顾性评估显示，具有良好的颅内疾病控制率（12周-24周内颅内疾病控制率约55%-65%）。塞瑞替尼、阿来替尼是第二代ALK抑制剂，其中塞瑞替尼在抗ALK的体外效能上是克唑替尼的20倍^[44]^。Gainor等^[45]^研究显示，ALK阳性肿瘤在LM患者（经克唑替尼、塞瑞替尼、化疗和放疗治疗后进展）中，3/4的患者在接受阿来替尼治疗后LM的放射学和神经学改善，而其余患者的中枢神经系统疾病稳定。劳拉替尼和布加替尼是被批准用于NSCLC的ALK抑制剂，一项Ⅰ期研究^[46]^结果中，脑脊液浓度与劳拉替尼血浆浓度的平均比值为0.75，表明劳拉替尼对中枢神经系统的渗透率较高。Felip等^[47]^报告，在两次或三次ALK抑制剂失败后，使用劳拉替尼治疗的患者的颅内客观反应持续时间为12.4个月，这表明使用多个ALK抑制剂有利于更好的结果。Yamamoto等^[48]^通过对1例复发性脑转移和脑膜播散并且多线治疗失败的NSCLC-ALK阳性患者回顾性分析中，通过5种ALK抑制剂的序贯治疗，患者获得了长达7年以上的生存期。

### 2.2 鞘内化疗

鞘内化疗（intrathecal chemotherpy, ITC）可以将抗肿瘤药物通过腰椎穿刺等方法直接注入脑脊液中，被认为是治疗脑膜转移癌的有效方法。正常的血-脑屏障及血-脑脊液屏障对大多数全身给药的抗肿瘤药物进入中枢神经系统起到了限制作用。因此，ITC的目标是跨过血-脑脊液屏障，使药物在脑脊液中的暴露增加，同时降低全身毒性，通过这种方法，可以使用较小的剂量获得更高的药物浓度^[49]^。

ITC治疗中甲氨蝶呤、阿糖胞苷和塞替派是常用的药物^[50]^。甲氨蝶呤是治疗脑膜转移癌经验最丰富的药物，但明确的给药时间和治疗持续时间暂未有统一结论。最常见的甲氨蝶呤剂量为10 mg-15 mg，每周2次，持续4周-6周，作为治疗诱导，如果诱导后细胞学检查呈阴性，则每周进行一次诱导治疗，持续1个月，然后转为每月维持ITC，有人建议，对于细胞学检查未清除且临床结果稳定或改善的患者，建议在转为维持性ITC前继续诱导性ITC治疗1个月，并在临床或脑脊液细胞学恶化的情况下，可能考虑终止ITC^[51]^。培美曲塞联合铂类药物目前被认为是晚期NSCLC，特别是非鳞状细胞恶性肿瘤患者的一线治疗方法^[52]^。尽管在血-脑屏障完整的患者中测得的培美曲塞脑脊液浓度较低，无法有效对抗中枢神经系统相关疾病^[53]^，但接受培美曲塞维持治疗的无脑转移患者发生的脑转移的概率少于其他治疗方案的患者^[54]^。在全球首个应用培美曲塞治疗EGFR-TKIs治疗后进展的肺腺癌脑膜转移癌的单臂Ⅰ期/Ⅱ期临床试验^[55]^（ChiCTR1800016615）中，前期研究确定了培美曲塞50 mg为NSCLC-LM患者鞘内注射的治疗剂量，Ⅱ期研究显示培美曲塞鞘内注射在多线靶向治疗失败肺癌脑膜转移癌取得很好的疗效（客观有效率超过80%），中位生存时间超过9个月，安全性也明显好于传统药物，更加证明了培美曲塞在ITC中的治疗潜力。

### 2.3 免疫治疗

在神经肿瘤学中，免疫系统起着重要作用^[56]^。传统印象中枢神经系统的特征之一是缺乏典型的淋巴引流系统，是肿瘤转移逃逸的免疫豁免器官，但是近年来随着基础科学进展发现颅内同样具有完善的淋巴系统，并且针对脑转移瘤进行免疫攻击^[57]^。免疫治疗在肺癌治疗中已经成为一线治疗的常用方案^[58-60]^。脑脊液中也存在相关免疫反应，因此，免疫检查点抑制剂（immune checkpoint inhibitors, ICIs）可以同时治疗原发性肿瘤和脑膜转移癌，使病灶缩小，同时改善神经症状和认知功能^[61]^。

Nivolumab促进抗肿瘤活性的机制是通过对T细胞上程序性死亡受体1（programmed cell death 1, PD-1）和程序性死亡配体1（programmed cell death ligand 1, PD-L1）激活的阻断，从而发挥免疫调节剂的作用。病例报道^[62, 63]^显示，接受Nivolumab治疗后，如幻听等临床体征有所缓解。并且在2例脑及脑膜转移患者行鞘内注射Nivolumab后，临床症状有所改善并且无明显不良反应^[64]^。Brastianos等^[65]^在ICIs在脑膜转移癌患者中的有效性和安全性的前瞻性临床试验中表示，Pembrolizumab可作为一种有效安全的方式，在部分对治疗选择有限的患者中使用，但试验研究规模小、受患者群体异质的影响，Pembrolizumab与其他疗法的联合治疗脑膜转移癌需进一步研究。

因为脑膜转移癌的诊断和疗效评价标准目前尚无国际统一标准，所以很难开展大规模临床试验，目前有几项单臂或含有少部分脑膜转移癌病例Ⅲ期研究结果发表，显示出免疫治疗对部分脑膜转移癌有一定疗效，但中位生存时间都很短，免疫治疗对于驱动基因阴性肺癌患者尤其是PD-L1高表达或高度微卫星不稳定（microsatellite instability-high, MSI-H）的脑膜转移癌可能是一种选择^[62, 66, 67]^，由于*EGFR*突变型肺癌脑膜转移癌患者驱动基因阳性患者占据更高的比例，在实体瘤的临床研究中对该类型患者采用ICIs治疗并不能取得良好的疗效，包括美国国立综合癌症网络（National Comprehensive Cancer Network, NCCN）等指南也不推荐免疫治疗用于驱动基因阳性肺癌前线应用免疫治疗，因此对于LM的免疫治疗仍需要更深入的基础及临床研究（表 1）。

**表 1:** 文献中的治疗效果 Therapeutic effects in the literature

Reference	Number of patients	Study design	Treatment	Results
Yang *et al*, 2020^[41]^	41	Phase Ⅰ; Prospective	Osimertinib	mOS: 11 mon PFS: 8.6 mon
Schuler *et al*, 2016^[36]^	81	Retrospective	Afatinib	PFS: 8.2 mon *vs* 5.4 mon
Hoffknecht *et al*, 2015^[37]^	573 (100 BM/LM)	Retrospective	Afatinib	mOS: 3.6 mon
Gainor *et al*, 2015^[45]^	4	Case report	Alectinib	Three of four patients experienced significant clinical and radiographic improvements in LM upon treatment with Alectinib
Yamamoto *et al*, 2021^[48]^	1	Case report	Ceritinib, Alectinib, Brigatinib and Lorlatinib	The overall time from the start of Crizotinib to Lorlatinib is 89.5 mon without relapse
Felip *et al*, 2021^[47]^	139	Phase Ⅱ; Prospective	Lorlatinib	mPFS: 6.6 mon mOS: 20.7 mon
Jackman *et al*, 2015^[35]^	7	Phase Ⅰ; Prospective	Erlotinib 750 mg (*n*=3) *vs* Erlotinib 1, 000 mg (*n*=4)	mOS: 3.5 mon
Clarke *et al*, 2010^[34]^	2	Case report	Erlotinib 1, 000 mg/wk-1, 500 mg/wk	Improved outcome for LM from NSCLC may be achieved by strategies that yield higher CSF levels of EGFR-TKIs
Fan *et al*, 2021^[55]^	30	Phase Ⅰ/Ⅱ; Prospective	ITC	mOS: 9 mon
ITC: intrathecal chemotherapy; BM: brain metastases; LM: leptomeningeal disease; mOS: median overall survival; mPFS: median progression-free survival; CSF: cerebrospinal fluid; EGFR-TKIs: epidermal growth factor receptor-tyrosine kinase inhibitors; NSCLC: non-small cell lung cancer.				

## 3 总结

肺癌脑膜转移癌因诊断率低、预后差，是目前临床的痛点和难点，如何提高脑膜转移癌脑脊液细胞学阳性率是影响治疗甚至可作为影响预后的一项重要因素。

综合已有的研究结果发现，具有临床症状及影像学阳性表现的临床诊断为脑膜转移癌的患者，腰穿操作过程中样本离体后快速固定处理是提高脑脊液细胞病理学阳性诊断的关键因素。对于确认为脑膜转移癌的患者，同时采用鞘内化疗联合靶向或者免疫治疗，可以延长脑膜转移癌患者的生存时间，提高其生活质量。
